# ERASURE: early autologous blood pleurodesis for postoperative air leaks—a randomized, controlled trial comparing prophylactic autologous blood pleurodesis versus standard watch and wait treatment for postoperative air leaks following thoracoscopic anatomic lung resections

**DOI:** 10.1186/s13063-023-07875-z

**Published:** 2024-01-02

**Authors:** Ioannis Karampinis, Christian Ruckes, Fabian Doerr, Servet Bölükbas, Sara Ricciardi, Giuseppe Cardillo, Carlos Galvez, Bogdan Vidmar, Tomaz Stupnik, Vincent Fang, Rene Horsleben Petersen, Eric Dominic Roessner

**Affiliations:** 1 Department of Thoracic Surgery, Centre for Thoracic Diseases, Mainz University, Mainz, Germany; 2 Interdisciplinary Center for Clinical Trials (IZKS Mainz), Mainz University, Mainz, Germany; 3 Department of Thoracic Surgery, University Medicine Essen-Ruhrlandclinic, Essen, Germany; 4 Department of Thoracic Surgery, Azienda Ospedaliera San Camillo Forlanini, Rome, Italy; 5 Department of Thoracic Surgery, Hospital General Universitario Alicante, Alicante, Spain; 6 Department of Thoracic Surgery, Ljubljana University Medical Centre, Ljubljana, Slovenia; 7 Department of Thoracic Surgery, Shanghai Chest Hospital, Shanghai, China; 8 Department of Cardiothoracic Surgery, University of Copenhagen, Copenhagen, Denmark

**Keywords:** Blood patch, Lobectomy, Segmentectomy, Lung surgery, Pleurodesis, Prolonged air leak

## Abstract

**Background:**

The prolonged air leak is probably the most common complication following lung resections. Around 10–20% of the patients who undergo a lung resection will eventually develop a prolonged air leak. The definition of a prolonged air leak varies between an air leak, which is evident after the fifth, seventh or even tenth postoperative day to every air leak that prolongs the hospital stay. However, the postoperative hospital stay following a thoracoscopic lobectomy can be as short as 2 days, making the above definitions sound outdated. The treatment of these air leaks is also very versatile. One of the broadly accepted treatment options is the autologous blood pleurodesis or “blood patch”. The purpose of this trial is to investigate the impact of a prophylactic autologous blood pleurodesis on reducing the duration of the postoperative air leak and therefore prevent the air leak from becoming prolonged.

**Methods:**

Patients undergoing an elective thoracoscopic anatomic lung resection for primary lung cancer or metastatic disease will be eligible for recruitment. Patients with an air leak of > 100 ml/min within 6 h prior to the morning round on the second postoperative day will be eligible for inclusion in the study and randomization. Patients will be randomized to either blood pleurodesis or watchful waiting. The primary endpoint is the time to drain removal measured in full days. The trial ends on the seventh postoperative day.

**Discussion:**

The early autologous blood pleurodesis could lead to a faster cessation of the air leak and therefore to a faster removal of the drain. A faster removal of the drain would relieve the patient from all the well-known drain-associated complications (longer hospital stay, stronger postoperative pain, risk of drain-associated infection, etc.). From the economical point of view, faster drain removal would reduce the hospital costs as well as the costs associated with the care of a patient with a chest drain in an outpatient setting.

**Trial registration:**

German Clinical Trials Register (DRKS) DRKS00030810. 27 December 2022

## Administrative information

**Table:** 

Title {1}	ERASURE Trial: early autologous blood pleurodesis for postoperative air leaks—a randomized, controlled trial comparing prophylactic autologous blood pleurodesis versus standard watch and wait treatment for postoperative air leaks following thoracoscopic anatomic lung resections-study protocol
Trial registration {2a and 2b}	German Clinical Trials Register (DRKS) DRKS-ID: DRKS00030810 Registration date: 27.12.2022 URL: https://drks.de/search/de/trial/DRKS00030810
Protocol version {3}	Approved protocol version V 2, 20.10.2022 by the local ethics committee. Due to changes in the participating centres and because the trial registration was completed after the ethical approval, minor changes have been made to the study protocol (currently V 3, 05.03.2023). Both study protocols will be available for review.
Funding {4}	Institutional resources will support the study.
Author details {5a}	^1^ Department of Thoracic Surgery, Centre for Thoracic Diseases, Mainz University, Germany ^2^ Interdisciplinary Center for Clinical Trials (IZKS Mainz), Mainz University, Germany ^3^ Department of Thoracic Surgery, University Medicine Essen-Ruhrlandclinic, Essen, Germany ^4^ Department of Thoracic Surgery, Azienda Ospedaliera San Camillo Forlanini, Rome, Italy ^5^ Department of Thoracic Surgery, Hospital General Universitario Alicante, Spain ^6^ Department of Thoracic Surgery, Ljubljana University Medical Centre. Slovenia ^7^ Department of Thoracic Surgery, Shanghai Chest Hospital, China ^8^ Department of Cardiothoracic Surgery, University of Copenhagen, Denmark
Name and contact information for the trial sponsor {5b}	Department of Thoracic Surgery, Centre for Thoracic Diseases, Mainz University, Germany Principal investigator: Prof. Eric Dominic Roessner, MD, FEBTS Co-investigator: Ioannis Karampinis, MD Department of Thoracic Surgery, Centre for Thoracic Diseases, Mainz University, Germany Langenbeckstr. 1, 55131 Mainz, Germany Email: Eric.roessner@unimedizin-mainz.de Ioannis.karampinis@unimedizin-mainz.de Tel: +49-6131-17-4602
Role of sponsor {5c}	There are no conflicting agreements with further sponsors or funders that would influence data acquisition, management, analysis or interpretation or dissemination of the results. The study investigators have exclusive access and control over all the aforementioned steps of the trial.

## Introduction

### Background and rationale {6a}

Every year, around 1.8 million people are diagnosed and 1.6 million die of lung cancer [1]. The morbidity associated with the disease is horrifying. In 2016, lung cancer was responsible for 36.4 million disability-adjusted life years (DALYs) [1]. The NLST trial showed a significant reduction in the mortality of lung cancer after routine screening with a low-dose CT scan of the chest [2]. However, the screening has not been broadly implemented yet, expecting that the rate of patients being diagnosed with early-stage lung cancer will substantially increase in the following years.

The most common complication following lung surgery is postoperative air leak. Air leaks result from incomplete closure of small airways during the division of lung tissue and can be monitored through the chest drain, which is placed at the end of an operation. Most air leaks heal within the first 24 h after a lung procedure. However, 10–20% of the operated patients will eventually develop a prolonged air leak [3].

The exact incidence of postoperative air leak widely varies, mainly due to the absence of a commonly accepted definition. The traditional definition of a prolonged air leak is an air leak, which lasts over 7 days. This definition is based on studies using analogue drain systems (underwater seal) to monitor the air leak. The expansion of video-assisted thoracoscopic surgery, the modern anaesthetic techniques and the evolution brought by the implementation of the ERAS pathway [4] have reduced the postoperative hospital stay after a lobectomy down to 2–3 days, making the definition of prolonged air leak clearly outdated. Furthermore, the modern electronic suction devices, which are nowadays used to monitor the air leak, enable a much more objective quantification of the air leak. It is therefore reasonable to expect that postoperative air leaks will soon become the most relevant complication in thoracic surgery.

The purpose of this trial is to investigate the impact of a prophylactic autologous blood pleurodesis in reducing the duration of the postoperative air leak and therefore the time till the drain can be removed.

### Objectives {7}

The hypothesis of the trial is that autologous blood pleurodesis will lead to a faster cessation of the air leak and prevent the air leak from becoming prolonged. This should lead to a shorter time that the drain needs to be kept in situ and therefore reduce the hospital stay, the drain-associated burden for the patient and the treatment costs.

### Trial design {8}

The ERASURE trial is an investigator-initiated, multicentre, prospective, randomized (1:1), controlled, open-label, parallel-group trial.

## Methods: participants, interventions and outcomes

### Study setting {9}

Patients will be referred to the trial centres for the treatment of primary or secondary lung lesions. All participating sites are either academic or university hospitals.

The following are the participating centres:

Department of Thoracic Surgery, Centre for Thoracic Diseases, Mainz University, Germany

Department of Thoracic Surgery, University Medicine Essen-Ruhrlandclinic, Essen, Germany

Department of Thoracic Surgery, LungenClinik Grosshansdorf, Grosshansdorf, Germany

Department of Thoracic Surgery, Azienda Ospedaliera San Camillo Forlanini, Rome, Italy

Department of Thoracic Surgery, Hospital General Universitario Alicante, Spain

Department of Thoracic Surgery, Ljubljana University Medical Centre. Slovenia

Department of Thoracic Surgery, Shanghai Chest Hospital, China

Department of Cardiothoracic Surgery, University of Copenhagen, Denmark

### Eligibility criteria {10}

#### Key inclusion criteria

Adult patients undergoing a scheduled thoracoscopic (video-assisted thoracoscopic surgery (VATS)) anatomic lung resection for primary or secondary lung tumours and presenting with an air leak > 100 ml/min within 6 h prior to the morning round of the second postoperative day will be eligible for enrollment.

#### Key exclusion criteria

The following are the key exclusion criteria:

1. Patients undergoing an open lobectomy
2. Patients undergoing complex lobectomies (bronchoplastic reconstructions, etc.)
3. Patients requiring invasive or positive pressure non-invasive ventilation except in the first 6 h following the operation
4. Patients undergoing re-operation during the same hospital admission
5. Suspected or proven bronchial stump leakage
6. Intraoperative use of sealants, pleural tents or talcum

All trial interventions will be performed either by surgeons or by surgical trainees.

### Who will take informed consent? {26a}

Informed consent will be obtained by the investigator (local principal investigator-consultant surgeon) or a person designated by the investigator in accordance with the current Good Clinical Practice Guideline and the Declaration of Helsinki. Prior to the beginning of the recruitment, each participating centre and coordinating investigator will be responsible for obtaining local institutional review board (IRB)/IEC approval of the written informed consent, study protocol and any other written information that will be provided to subjects.

### Additional consent provisions for collection and use of participant data and biological specimens {26b}

Participant data will be handled in accordance with the current Good Clinical Practice Guideline and the Declaration of Helsinki. No biological specimens will be obtained in this trial.

### Interventions

#### Explanation for the choice of comparators {6b}

Patients who enter the control group will be treated conservatively. The electronic suction device will be set on gravity mode (− 8 cmH_2_O or − 0.8 kPa). No interventions are allowed in order to speed up the healing of the air leak. This is the routine way to deal with postoperative air leaks in most thoracic centres worldwide. After the seventh postoperative day, patients with persisting air leak will be treated according to each participating centre’s preference. In case the air leak stops before the seventh postoperative day, the drain will be removed accordingly.

#### Intervention description {11a}

Patients who meet the eligibility criteria will be randomized. Patients who enter the interventional group will receive an autologous blood pleurodesis. A sterile bedside set-up will be created for the procedure. A leur-lock connector will be attached to the chest drain in a sterile manner; 100–120 ml of patient blood will be obtained from a peripheral or central venous line. The blood will be then administered through the leur lock connector into the chest drain. The chest drain will be then flushed with 20 ml of sterile normal saline to avoid clotting of the drain. The tubing of the electronic suction system will be then raised 60 cm above the level of the patient. The patient will be asked to change places and move while in bed in order for the blood to spread equally in the pleural cavity. After the 2 h, the patient will be free to mobilize and the electronic suction system will be turned on and set on gravity mode (− 8 cmH_2_O or − 0.8 kPa). In case the air leak persists, the procedure will be repeated on the third postoperative day. After that, no further interventions are scheduled till the seventh postoperative day. After the seventh postoperative day, patients with persisting air leak will be treated according to each participating centre’s preference. In case the air leak stops before the seventh postoperative day, the drain will be removed accordingly (regardless of the amount of fluid drained).

#### Criteria for discontinuing or modifying allocated interventions {11b}

Any modifications that need to be applied on the chest drain (e.g. patient’s condition requires change in the suction or other intervention) will be considered interventions and documented on the electronic case report form. Cross-over is not permitted before the seventh postoperative day, when the formal trial participation ends. After the seventh postoperative day, any type of treatment is permitted, as it is no longer associated with the trial.

#### Strategies to improve adherence to interventions {11c}

In order to improve adherence to the intervention protocol, we have created a standard operating operating procedure (SOP) for the autologous blood pleurodesis.

#### Relevant concomitant care permitted or prohibited during the trial {11d}

No changes to the drain suction, manipulations on the drain (e.g. pulling back the drain) or any other ways to influence an air leak are permitted during the trial.

#### Provisions for post-trial care {30}

Not applicable. Autologous blood pleurodesis is a well-established method for treating postoperative air leaks with a very low rate of adverse events. Patients developing complications following autologous blood pleurodesis will be treated according to the local investigators’ preference. No compensation will be provided.

### Outcomes {12}

#### Primary endpoint

Time to drain removal measured in full days

#### Secondary endpoints

The following are the secondary endpoints:

1. Time to cessation of the air leak (calculated in postoperative hours)
2. Length of postoperative stay measured in full days
3. Rate of (redo) interventions due to persistent air leaks
4. Rate of pleural empyemas

#### Outcome measures

Time to drain removal measured in full days was chosen as the primary endpoint for several reasons:

(1) The intervention of this trial is a locally applied therapy enabling a more effective and faster sealing of the postoperative air leak. The efficacy of this treatment is more accurately evaluated by the time to drain removal. (2) It reflects the success or failure of the treatment directly, since removing the drain implies the cessation of the postoperative air leak. (3) It is a parameter, which is unique and irreversible throughout the patients’ treatment and therefore not susceptible to interpretation bias from the medical team. (4) The parameter is clinically relevant and practical to use. The main reason is that chest drains are not usually removed overnight. Likewise, patients are usually not discharged overnight. (5) Several studies have analysed the efficacy of autologous blood pleurodesis using the time to drain removal as the primary efficacy endpoint making it a widely accepted parameter to use as a primary outcome measure [5, 6]. The secondary outcomes include all relevant perioperative and patient-reported outcomes in thoracic surgery trials using widely accepted definitions. In order to assess the variability of the drain removal protocols among the participating centres, the shortest potential drainage time in postoperative hours will be calculated by retrieving the relevant data from the electronic suction device memory. Furthermore, in order to assess further complications associated with the procedure and potentially with the intervention (using the Clavien-Dindo grading system [7], the length of the postoperative stay calculated in full days will be evaluated. The rate of re-interventions due to persistent air leak after the initial removal of the drain will be also measured in order to detect treatment failures. The pleural empyema is the only well-documented complication of autologous blood pleurodesis with an incidence of 1.5% [8].

### Participant timeline {13}

**Fig. 1:**
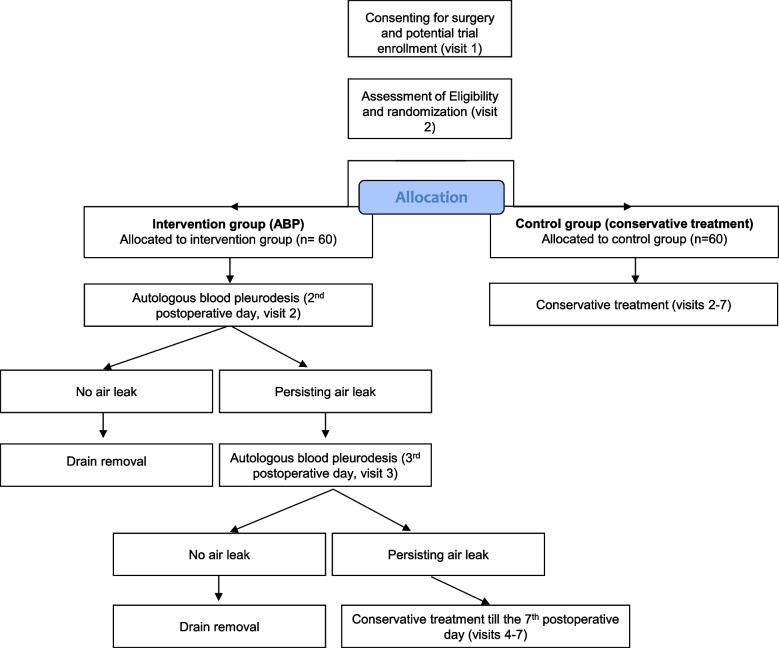
Trial flowchart

Screening for eligibility will take place in the outpatient clinic (Fig. 1 and Table 1). Patients will be consented for the trial. Patients who undergo a thoracoscopic, anatomic lung resection and still have an air leak > 100 ml/min within 6 h before the morning ward round on the second postoperative day will be randomized.

**Table 1:** Trial timeline

	Baseline	Randomization	Follow-up				
	Visit 1	Visit 2	Visit 3	Visit 4	Visit 5	Visit 6	Visit 7
	OPC	POD 2	POD 3	POD 4	POD 5	POD 6	POD 7
Baseline data	x	x					
Informed consent	x						
Eligibility	x	x					
Intraoperative data		x					
Intervention		x/0	x/0				
Clinical events, complications		x	x	x	x	x	x
SAE		x	x	x	x	x	x

*OPC* outpatient clinic, *POD* postoperative day, *SAE*: serious adverse events, *x* shows when a specific step of the trial will take place, *x/0* shows if the intervention will be performed depending on the trial group

Patients who are randomized in the intervention group will receive an autologous blood pleurodesis on the same day and if the air leak does not resolve, on the third postoperative day. After that, no further interventions are permitted unless the clinical condition of the patient requires deviation from the trial. Patients who are randomized in the control group will be treated according to the watchful waiting strategy until the seventh postoperative day. Trial participation ends on the seventh postoperative day, and every patient can be treated according to the preferences of the participating centre.

### Sample size {14}

The sample size calculation considered the median drainage removal times of the study by Shackcloth et al. [9]. This seems to be the study of the highest quality on this topic so far. Shackcloth reported drainage removal times of 6.5 days in the control group and 12.0 days in the intervention group. The maximum observation time was set to 14 days, and no accrual time is considered. For the sample size calculation, we assumed a difference in the removal times of 2 days (compared with the 5.5 days reported by Shackcloth et al.). This is much more conservative and reflects more on the current surgical practice than 18 years ago, when Shackcloths’ trial was published.

The sample size calculation was based on median drainage removal times of 4 days in the control group and 2 days in the experimental group. The maximum observation time was set to 7 days, and no accrual time is considered. With a two-sided level of significance of 5% and a power of 90%, a sample size of 110 (= 2 × 55) will be needed using a log-rank test for planning. When considering slightly less than 10% of dropouts, 120 patients (= 2 × 60) should be randomized. The sample size was calculated with SAS version 9.4.

### Recruitment {15}

Anatomic lung resections belong to the most commonly performed thoracic procedures. The incidence of the prolonged air leak using the period of 7 days to define ‘prolonged’ is 10–20% [3]. Other studies report even higher incidence than that. These data are based on older studies, where the air leak was measured with the traditional underwater seal systems. The new electronic suction devices, which will be used in this trial, are far more sensitive in identifying and quantifying air leaks than the traditional underwater seal. This study will assess the role of autologous blood pleurodesis as a prophylactic treatment by applying autologous blood pleurodesis on the second and third postoperative days. It is therefore rather conservative to assume that 20% of the screened patients will have an air leak > 100 ml/min on the first postoperative day. This will result in 710 patients needing to be screened for this trial. This trial will be performed among seven high-volume institutions. At each institution over 200 patients are treated for primary lung cancer every year. This translates into approximately *n* = 1500 patients that will be screened during the enrolment period of 12 months. After the exclusion of patients not meeting the eligibility criteria and those declining participation in the study, a sufficient number of patients will remain eligible for inclusion. In fact, just two patients need to be enrolled per centre every month to meet the anticipated enrolment period, which appears feasible. Furthermore, all centres are academic institutions with a dedicated trial centre ensuring the screening of all scheduled patients and inclusion of all eligible patients.

### Assignment of interventions: allocation

#### Sequence generation {16a}

Computer-generated allocation, no stratification planned. All trial patients will be randomized after confirmation of eligibility on the second postoperative day.

#### Concealment mechanism {16b}

The randomization process is implemented in the electronic case report form, and the sequence is only generated after the investigator has confirmed the eligible for every included patient.

#### Implementation {16c}

A computerized tool that is embedded in the electronic case report form will generate the randomization sequence (1:1 randomization).

### Assignment of interventions: blinding

#### Who will be blinded {17a}

Open-label trial, no blinding.

#### Procedure for unblinding if needed {17b}

Not applicable.

### Data collection and management

#### Plans for assessment and collection of outcomes {18a}

Patient data will be assessed and collected by the local surgical team and will be entered in the electronic case report form. Responsible for the data accuracy is the local primary investigator. The electronic case report form is designed to request an electronic signature from the investigator after having entered any type of data. The pseudonymization of the data will be performed locally at each participating centre. Each centre will have full access to its own data. The principal investigator and the co-investigator will have full access to the complete pseudonymized dataset. A summary of the data management and confidentiality has been included both in the trial protocol and in the consent form that will be handed over to the study participants. Training of the investigators is scheduled before the beginning of the trial in order to ensure correct data entry.

#### Plans to promote participant retention and complete follow-up {18b}

The participation in this trial and all associated interventions and data acquisition are completed during the hospital stay of the patients. A high adhesion to the trial interventions and low rate of “loss to follow-up” are to be expected.

#### Data management {19}

Data will be entered electronically in the electronic case report form (eCRF) The electronic case report form is an online platform, which has been specifically set up by the Interdisciplinary Center for Clinical Trials (IZKS Mainz) for the purpose of this specific trial. An electronic signature request has been embedded in several levels of the eCRF and is required in order to proceed throughout the eCRF. Errors will be detected by programmes, which are designed to detect missing data or deviations from the expected outcomes/values. The process of data extraction, analysis and dissemination will only include pseudonymized data. The Interdisciplinary Center for Clinical Trials (IZKS Mainz) will be responsible for data management and data archive. Data will be stored in a pseuodonymized form for 25 years. All data specified in the trial protocol will be documented in a clinical database. The investigator or the designated representatives are obliged to clarify and solve queries. If no further corrections are to be made in the database, it will be locked and used for statistical analysis.

#### Confidentiality {27}

Electronic data will be kept confidential and not accessible to any unrelated person or third party throughout the whole trial. The pseudonymization process will be performed locally at each participating centre. The coordinating investigator of each participating centre will be responsible for the confidentiality of the pseudonymization and the adherence to the Good Clinical Practice guidelines.

All data management procedures at the coordinating centre will be conducted according to written defined standard operating procedures (SOPs) of the IZKS that guarantee an efficient conduct complying with Good Clinical Practice (GCP) and under strict observance of national and EU regulations. Source data are to be stored for at least 10 years after trial termination in the centre archive. At the end of the study, the data will be transformed into different data formats for archiving to ensure that it can be reused.

##### Dissemination of results

The trial has been registered at the German Clinical Trials Registry (DRKS) and the trial protocol will be published. International guidelines such as SPIRIT and CONSORT will be strictly adhered to. Results will be presented at national and international conferences. Publication in international peer-reviewed journals is intended.

#### Plans for collection, laboratory evaluation and storage of biological specimens for genetic or molecular analysis in this trial/future use {33}

N/A, no biological specimens will be collected as part of this trial.

## Statistical methods

### Statistical methods for primary and secondary outcomes {20a}

The primary endpoint (the time to drain removal in full days) will be analysed within a Cox regression model with treatment as a fixed factor and centre as a covariate. Model assumptions will be checked by Schoenfeld residuals. The two-sided significance level is set to *α* = 5%. The primary analysis population is the ITT population consisting of all randomized patients. Treatment differences will be displayed by the estimate of the hazard ratio and the 95% confidence interval. Sensitivity analyses will be done by adding additional variables like smoking (yes/no) and sex in the regression model, although sex is not known as an influencing factor for time to drain removal.

Safety: Absolute and relative frequencies of adverse events.

### Interim analyses {21b}

No interim analysis is planned.

### Methods for additional analyses (e.g. subgroup analyses) {20b}

Secondary endpoints: The length of postoperative stay will be analysed by the same model as the primary analysis. The rate of re-interventions and pleural empyemas will be analysed by a logistic regression model.

### Methods in analysis to handle protocol non-adherence and any statistical methods to handle missing data {20c}

Missing data will not be replaced by imputation or any other similar methods.

### Plans to give access to the full protocol, participant-level data and statistical code {31c}

The study has been registered on a nationwide register. A website has been created for the purposes of the trial (https://www.unimedizin-mainz.de/zft/erasure.html). The full study protocol, information for patients and the ethical approval are available on the website of the trial. The trial dataset will remain confidential until the final analysis has been performed and published. The main dataset will be stored in a way that allows further sharing and pooling of the metadata with other research groups. The data will not be accessible to anyone unauthorized. The principal investigator in agreement with the Interdisciplinary Center for Clinical Trials (IZKS Mainz) can only provide access to the data upon formal written request. In case of conflicting requests to use data for purposes other than those approved by the Ethics Committee, the Ethics Committee will be contacted. Data sharing will be performed in accordance with the principles outlined in the Good Practice Principles for Sharing Individual Participant Data from Publicly Funded Clinical Trials.

### Oversight and monitoring

#### Composition of the coordinating centre and trial steering committee {5d}

The coordinating centre is composed of the principal investigator, the co-investigator and one study nurse. This group together with the coordinating investigator of each participating centre or another person designated by the coordinating investigator will meet at least once a month during the first 3 months of the trial to ensure the smooth progress of the trial and solve any related issues. A representative from the Interdisciplinary Center for Clinical Trials (IZKS Mainz) will participate in these meetings if necessary. Depending on the course of the trial, these meetings will take place once every 2 months after the first three months of the trial.

#### Composition of the data monitoring committee, its role and reporting structure {21a}

No formal trial site monitoring is planned for this trial. Data reporting will be controlled by the Interdisciplinary Center for Clinical Trials (IZKS Mainz) in order to ensure protocol adherence, quality and integrity of the data collection, monitoring of the adverse events and compliance with the reporting protocol.

#### Adverse event reporting and harms {22}

Serious adverse events will be directly reported to the principal investigator. Depending on causality and frequency, the principal investigator along with the local investigator and the coordinating team will assess the need for further actions (addressing the issue to the local or the leading ethics committee, depending on the frequency of the serious adverse event potential premature closure of the trial).

#### Frequency and plans for auditing trial conduct {23}

The recruitment status of the trial will be audited once monthly in order to determine the progress of the trial and potential changes that need to be made.

#### Plans for communicating important protocol amendments to relevant parties (e.g. trial participants, ethical committees) {25}

Any major modifications of the study protocol that affect the conduct of the trial or patient safety, risks or benefits including any changes in the study design, sample size, procedures or objectives will require a formal amendment of the trial protocol. Formal amendments to the trial protocol will be formally addressed to the leading ethics committee and after approval to the local ethics committees of the participating centres. Major modifications of the study protocol will be applied only after approval by the ethics committee.

#### Dissemination plans {31a}

The trial has been registered at an international trial registry, and the trial protocol will be published. International guidelines such as SPIRIT [10, 11] and CONSORT [12] will be strictly adhered to. Results will be presented at national and international conferences. Publication in international peer-reviewed journal is intended.

## Discussion

The widespread of minimally invasive thoracic surgery and the increasing implementation of the ERAS guidelines have led to a significant reduction in postoperative hospital stays in patients undergoing lung resections. The current length of hospital stay for a thoracoscopic lobectomy is around 4 days with some clinics discharging patients as early as on the first or second postoperative day. On the other hand, the definition of a prolonged air leak varies between the fifth and the tenth postoperative day down to any air leak that prolongs the hospital stay. It is therefore imperative to redefine prolonged air leaks. Resetting the definition will subsequently lead to surgeons being urged to deal with these air leaks sooner and this will hopefully lead to chest drains being removed earlier, patients having less discomfort, being discharged earlier and hospital costs being reduced.

One of the main concerns of autologous blood pleurodesis is its historical connection with the pleural empyema. This is the mostly reported complication of this procedure. Our recent meta-analysis estimated the empyema rate following an autologous blood pleurodesis at 1.5% [8]. However, it should be considered that the studies included in the meta-analysis reported outcomes on autologous blood pleurodesis that was performed on the seventh postoperative day or even later. It is well known that the later the procedure is performed, the higher the risk of infection (and consequently of empyema) is. It is therefore reasonable to expect that the true incidence of empyema following a prophylactic blood pleurodesis is well under 1%, which is medically acceptable. Furthermore, the drain itself harbours a certain risk of infection like every other foreign body penetrating the skin. The longer the drain stays, the higher the chances of a patient developing a drain-related infection. The empyema incidence for chest drains has not been reported, but it is probably comparable with the empyema rate following autologous blood pleurodesis. Based on these assumptions, we do not expect that performing autologous blood pleurodesis will be associated with more empyemas than those that occur due to a chest drain that needs to stay in for a longer period, due to a persisting air leak.

Autologous blood pleurodesis is an established and effective treatment for prolonged air leaks when the source of the air leak is the lung tissue and not leakage from the transected major airways. The purpose of this trial is to investigate the use of this modality in a prophylactic manner, in particular before the air leak becomes prolonged. This setting has several advantages compared with the old-fashioned blood patch. First, the early application on the second and third postoperative day enables a much earlier treatment of the air leak with all the aforementioned benefits. Second, it has several advantages from the infectiological point of view, since it is performed earlier, through a drain that has not been in situ for several days. Finally, yet importantly, it allows an earlier selection of the non-responders leading to a faster “step up” of the treatment.

## Trial status

The recruitment for the ERASURE trial has not begun at the time of the submission and is expected to begin on 01.05.2023. We expect to recruit the required 120 patients within 1 year after the beginning of the recruitment. The currently used version of the study protocol is version 3.0, which includes minor changes compared to the approved version 2.0.

## Data Availability

Only the members of the study group will have access to the final trial dataset. The dataset will be kept confidential till dissemination of the results.

## Abbreviations

VATS: Video-assisted thoracoscopic surgery
DALY: Disability-adjusted years
ERAS: Enhanced recovery after surgery
IRB: Institutional review board
IEC: Institutional ethics committee
eCRF: Electronic case report form
SOP: Standard operating procedure
GCP: Good Clinical Practice
OPC: Outpatient clinic
POD: Postoperative day
SAE: Serious adverse events
SPIRIT: Standard Protocol Items: Recommendations for Interventional Trials
CONSORT: Consolidating Standards of Reporting Trials
